# Dual sgRNA‐directed gene deletion in basidiomycete *Ganoderma lucidum* using the CRISPR/Cas9 system

**DOI:** 10.1111/1751-7915.13534

**Published:** 2020-01-20

**Authors:** Ke Liu, Bin Sun, Hao You, Jun‐Liang Tu, Xuya Yu, Peng Zhao, Jun‐Wei Xu

**Affiliations:** ^1^ Faculty of Life Science and Technology Kunming University of Science and Technology Kunming 650500 China

## Abstract

*Ganoderma lucidum* is an important medicinal mushroom in traditional Chinese medicine. However, the lack of adequate genetic tools has hindered molecular genetic research in and the genetic modification of this species. Here, we report that the presence of an intron is necessary for the efficient expression of the heterologous phosphinothricin‐resistance and green fluorescent protein genes in *G. lucidum*. Moreover, we improved the CRISPR/Cas9‐mediated gene disruption frequency in *G. lucidum* by adding an intron upstream of the Cas9 gene. Our results showed that the disruption frequency of the orotidine 5’‐monophosphate decarboxylase gene (*ura3*) in transformants containing the glyceraldehyde‐3‐phosphate dehydrogenase gene intron in the Cas9 plasmid is 14–18 in 10^7^ protoplasts, which is 10.6 times higher than that in transformants without any intron sequence. Furthermore, genomic fragment deletions in the *ura3* and GL17624 genes were achieved via a dual sgRNA‐directed CRISPR/Cas9 system in *G. lucidum*. We achieved a *ura3* deletion frequency of 36.7% in *G. lucidum*. The developed method provides a powerful platform to generate gene deletion mutants and will facilitate functional genomic studies in *G. lucidum*.

## Graphical abstract

An improved CRISPR/Cas9 system that contained the intron from the glyceraldehyde‐3‐phosphate dehydrogenase gene (*gpd*) for gene disruption in *G. lucidum* is described. An effective platform for target gene deletion was first established using a dual sgRNA‐directed CRISPR/Cas9 system in *G. lucidum*.

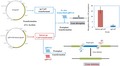

## Introduction

Medicinal mushrooms are rich sources of pharmacologically active compounds (Chaturvedi *et al.*, 2018). *Ganoderma lucidum*, a traditional medicinal mushroom, has been used to improve health and longevity in China for several millennia (Hsu and Cheng, 2018). These mushrooms exhibit several therapeutic activities, such as antitumor, immunomodulatory and antihypertensive activities (Russell and Paterson, 2006). Due to its wide range of pharmacological activities, *Ganoderma* has drawn widespread interest in recent years. The genomes of *Ganoderma* strains have recently been sequenced (Chen *et al.*, 2012; Kües *et al.*, 2015); however, functional characterization of the genes of interest is time‐consuming due to the lack of adequate molecular genetic tools.

Genetic transformation systems, including *Agrobacterium tumefacien*‐mediated and polyethylene glycol (PEG)‐mediated transformations, have been developed in *G. lucidum* (Shi *et al.*, 2012; Xu *et al.*, 2012; Xu and Zhong, 2015) and have been successfully used to enhance the production of bioactive compounds, such as ganoderic acids and polysaccharides (Zhou *et al.*, 2014; Li *et al.*, 2016; Zhang *et al.*, 2017a, 2017b; Fei *et al.*, 2019; Xu *et al.*, 2019). Gene silencing systems have also been used for the partial suppression of genes of interest in *G. lucidum* (Mu *et al.*, 2012; Liu *et al.*, 2018; Zhu *et al.*, 2019). So far, however, targeted gene deletion has not been established in *Ganoderma*, although it would be a powerful tool for *G. lucidum* research in the post‐genomic era.

Generally, homologous recombination (HR)‐mediated gene deletion occurs at a low frequency, because DNA integration mostly occurs ectopically through non‐homologous end joining (NHEJ) in filamentous fungi, animals and plants (Kück and Hoff, 2010). Although the frequency of HR is higher in NHEJ‐deficient filamentous fungi such as *Neurospora*, *Coprinopsis cinerea* and *Pleurotus ostreatus* (Ninomiya *et al.*, 2004; Nakazawa *et al.*, 2011; Salame *et al.*, 2012)*,* such strains may show growth defects, genomic instability and a low regeneration rate of protoplasts (de Jong *et al.*, 2010; Zhang *et al.*, 2011). Therefore, the development of novel molecular tools is required for efficient gene deletion in filamentous fungi.

The clustered regularly interspaced short palindromic repeats (CRISPR)/CRISPR‐associated protein 9 (Cas9) system has been successfully applied to genome editing in animals, plants and microbes (Arazoe *et al.*, 2015; Bortesi *et al.*, 2016; Li *et al.*, 2019; Schultz *et al.*, 2019). Recently, the CRISPR/Cas9 system has been used for the disruption of genes in higher fungi such as *Coprinopsis cinerea*, *Cordyceps militaris* and *Schizophyllum commune* (Sugano *et al.*, 2017; Chen *et al.*, 2018; Vonk *et al.*, 2019). Qin *et al.* reported the disruption of the *ura3* gene of *G. lucidum* and *G. lingzhi*, which revealed the potential of the CRISPR/Cas9 system for disrupting the genes of *Ganoderma* (Qin *et al.*, 2017). However, the disruption of the target gene must be highly efficient to facilitate genome editing approaches in *Ganoderma*. NHEJ‐mediated gene disruption using the CRISPR/Cas9 system typically produces small insertions and deletions in the target genes. This method usually lacks the ability to disrupt the function of regulatory sequences or elements in the non‐coding genome, whereas gene deletion is an effective alternative for achieving these aims (Cai *et al.*, 2018). Recently, dual sgRNA‐directed gene deletion using the CRISPR/Cas9 system has also been demonstrated in some species, such as humans, rabbits, *Caenorhabditis elegans* and *Arabidopsis* (Chen *et al.*, 2014; Zheng *et al.*, 2014; Song *et al.*, 2016; Durr *et al.*, 2018). However, there has been no report of dual sgRNA‐directed deletion of target genes in *Ganoderma* spp.

In this study, we show that the presence of an intron is necessary for the efficient expression of the heterologous phosphinothricin‐resistance gene (*bar*) and the green fluorescent protein gene (*gfp*) in *G. lucidum*. We describe an improved CRISPR/Cas9 system that contains an intron of the glyceraldehyde‐3‐phosphate dehydrogenase gene (*gpd*) for gene disruption in *G. lucidum*. Moreover, we demonstrate that the dual sgRNA‐directed CRISPR/Cas9 system is an efficient tool for gene deletion in *G. lucidum*. This technology provides a useful platform in basic and applied research for gene deletion in medicinal mushrooms.

## Results

### Expression of the heterologous genes *bar* and *gfp* in *G. lucidum*

**Figure 1:**
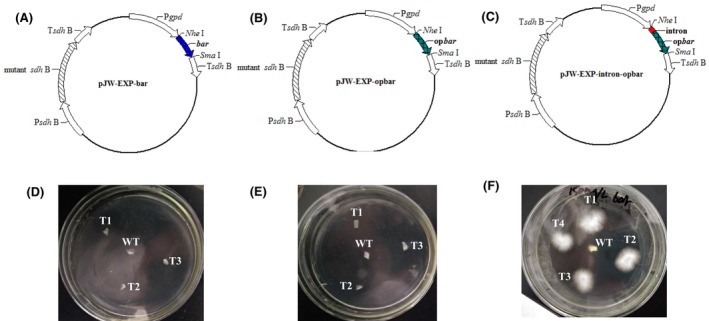
Selection of carboxin‐ and phosphinothricin‐resistant transformants on a selective CYM plate. Plasmid pJW‐EXP‐bar (A), pJW‐EXP‐opbar (B) and pJW‐EXP‐intron‐opbar (C). Selection of carboxin‐resistant pJW‐EXP‐bar transformants (D), pJW‐EXP‐opbar transformants (E) and pJW‐EXP‐intron‐opbar transformants (F) on a selective CYM plate with 150 mg l^−1^ phosphinothricin.

**Figure 2:**
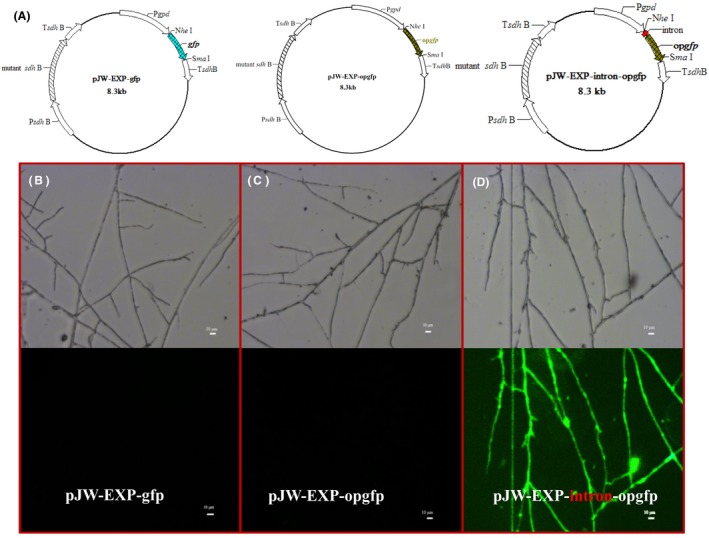
Expression of GFP in *G. lucidum* transformants. Plasmids pJW‐EXP‐bar, pJW‐EXP‐opbar and pJW‐EXP‐intron‐opbar (A). Phase‐contrast and fluorescence image of mycelium containing the plasmids pJW‐EXP‐bar (B), pJW‐EXP‐opbar (C) and pJW‐EXP‐intron‐opbar (D).

To efficiently express the Cas9 gene, we first evaluated the effects of codon optimization and intron addition on the expression of heterologous genes in *G. lucidum*. Currently, only the carboxin‐resistance gene *cbx* and hygromycin B‐resistance gene *hph* have proven useful as selection markers for the genetic manipulation of *G. lucidum*. In order to explore more genetic selection markers, the expression of *bar*, which encodes the phosphinothricin‐resistance gene, was investigated in *G. lucidum*. Plasmids pJW‐EXP‐bar, pJW‐EXP‐opbar and pJW‐EXP‐intron‐opbar (Fig. 1), which carry the *bar* gene (*opbar*), the codon‐optimized *bar* gene, and the 5′ intron of *G. lucidum gpd* with *opbar*, were constructed and transformed into protoplasts of *G. lucidum*, respectively. After the genetic transformation of *G. lucidum*, protoplasts with pJW‐EXP‐bar and pJW‐EXP‐opbar were unable to grow on the phosphinothricin‐containing CYM plate. However, four carboxin‐ and phosphinothricin‐resistant colonies were obtained with pJW‐EXP‐intron‐opbar (Fig. 1). These results indicated that the extra intron at the 5′ end of the phosphinothricin‐resistance gene is required for its efficient expression in *G. lucidum*. To confirm these results, we also constructed plasmid pJW‐EXP‐gfp, pJW‐EXP‐opgfp and pJW‐EXP‐intron‐opgfp (Fig. 2A) and transformed them into the protoplast of *G. lucidum*. Transgenes from carboxin‐resistant colonies were selected by PCR and subsequently microscopically screened for GFP expression. No fluorescence was detected in any of the carboxin‐resistant pJW‐EXP‐gfp and pJW‐EXP‐opgfp transformants (Fig. 2B and 2), despite confirmation of the presence of *gfp* and *opgfp* by PCR (data not shown). In contrast, all pJW‐EXP‐intron‐opgfp transformants exhibited green fluorescence in the *G. lucidum* mycelia (Fig. 2D). GFP expression was shown to be stable across five rounds of subculturing (data not shown). These results confirmed that a 5′ intron was essential for GFP expression in *G. lucidum*.

### Disruption of *ura3* in the engineered *G. lucidum* pJW‐EXP‐intron‐opcas9

**Figure 3:**
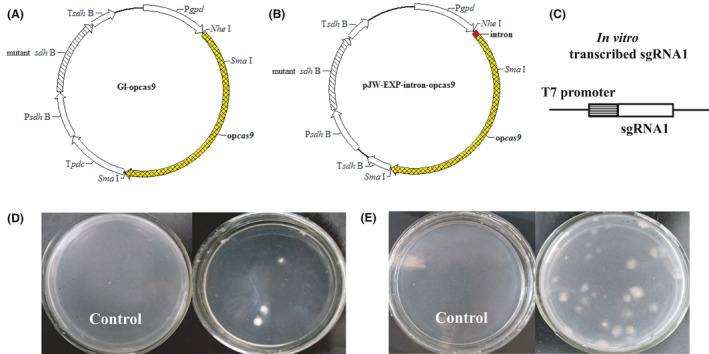
Selection of 5‐FOA‐resistant mutants on a selective MM plate. The design of plasmids GL‐opcas9 (A), pJW‐EXP‐intron‐opcas9 (B) and in *vitro*‐transcribed sgRNA1 (C). Selection of 5‐FOA‐resistant mutants after sgRNA1 targeting *ura3* was delivered into the transformants GL‐opcas9 (D) and pJW‐EXP‐intron‐opcas9 (E) on a selective MM plate.

To disrupt *ura3* in *G. lucidum*, plasmids GL‐opcas9 and pJW‐EXP‐intron‐opcas9 (Fig. 3A and 3) were first constructed and transformed into the protoplasts of *G. lucidum*, separately. After screening on selective CYM plates with 2 mg/L carboxin, and confirmation via genomic PCR analysis and sequencing, we obtained the stable transformants GL‐opcas9 and pJW‐EXP‐intron‐opcas9 for gene disruption. No morphological difference was observed between the transformants GL‐opcas9 and pJW‐EXP‐intron‐opcas9, and the growth rates of both transformants were similar (Fig. S1). *In vitro*‐transcribed sgRNA1 (100 µg) targeting *ura3* (Fig. 3C) was delivered into transformants GL‐opcas9 and pJW‐EXP‐intron‐opcas9, and 1‐3 and 28‐36 transformants resistant to 5‐fluoroorotic acid (FOA) were obtained per 2 × 10^7^ protoplasts, respectively (Fig. 3). The average disruption efficiency of *ura3* in pJW‐EXP‐intron‐opcas9 was 16 in 10^7^ protoplasts, which is 10.6 times higher than that in GL‐opcas9 (1.5 in 10^7^ protoplasts). The obtained *ura3* disruption frequency in GL‐opcas9 was comparable to that (0.2–1.78 in 10^7^ protoplasts) reported by Qin *et al.* (2017). Thirty 5‐FOA‐resistant colonies from the transformant pJW‐EXP‐intron‐opcas9 were selected, and transformation was confirmed using genome PCR with primers ura3‐F/ura3‐R and sequence analysis. These results are summarized in Table 1 and Data S3. The sequences of *ura3* of the wild‐type strain (WT) and four randomly selected 5‐FOA‐resistant colonies are shown in Fig. S2. The replacement, deletion and insertion sites were located 3 bp upstream of the protospacer‐adjacent motif (PAM) sequence, which indicated that DNA double‐strand breaks (DSBs) were directed by CRISPR/Cas9 and repaired via NHEJ. When sgRNA2 (100 µg) targeting *ura3* (Data S4) was delivered into pJW‐EXP‐intron‐opcas9, 28 transformants were obtained in 5‐FOA‐containing minimal medium (MM) plates. In *Ashbya gossypii*, different editing efficiency between the three *ADE2* sgRNA‐dDNAs was also found (Jimenez *et al.*, 2019). The observed difference in the disruption efficiency of *ura3* may be due to the fact that the Cas9‐generated alleles display sequence‐dependent bias (Allen *et al.*, 2019). These results are summarized in Data S4. The selected transformants contain deletions and insertions close to the PAM sequence of *ura3*. In contrast, no transformants were observed in the 5‐FOA‐containing MM plates without sgRNA1 or sgRNA2 from the control transformation of transformant pJW‐EXP‐intron‐opcas9. Our results demonstrated that both sgRNA1 and sgRNA2 are effective in disrupting *ura3* in the engineered *G. lucidum* pJW‐EXP‐intron‐opcas9.

**Table 1:** The mutation efficiency of insertion, deletion and replacement of *ura3.*

Mutant of *ura3*	Number of mutants	Efficiency (%)
Disruption	30	100
Insertion	25	83.3
Deletion	4	13.3
Replacement	1	3.3

sgRNA1 targeting *ura3* was delivered into protoplasts of *G. lucidum* transformant pJW‐EXP‐intron‐opcas9. Thirty individual 5‐FOA‐resistant mutants were assessed for insertion, deletion and replacement efficiency using the PCR and TA‐cloning sequence assay.

### Dual sgRNA‐directed deletion of *ura3* in the engineered *G. lucidum* pJW‐EXP‐intron‐opcas9

**Figure 4:**
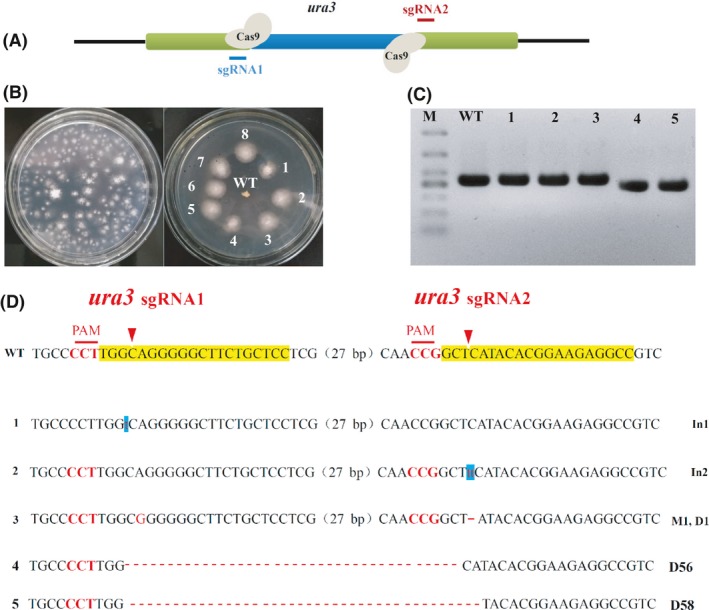
Dual sgRNA‐directed deletion of *ura3* in *G. lucidum*. A. Schematic representation of *ura3* of *G. lucidum* and sequences targeted by Cas9. B. Screening and re‐selection of 5‐FOA‐resistant mutants after sgRNA1 and sgRNA2 targeting *ura3* were delivered into transformant pJW‐EXP‐intron‐opCas9. C. Determination of the deletions of *ura3* in *G. lucidum* transformants by PCR. D. TA‐cloning of the target sites for each sgRNA in selected transformants. The sgRNA‐guiding sequences are highlighted in yellow. WT, the wild‐type strain; 1, 2, 3, 4, 5, *G. lucidum* transformants; M, DL500 DNA marker; In1 and In2, 1bp insertion and 2 bp insertion; M1, 1 bp replacement; D1, D56 and D58, 1 bp deletion, 56 bp deletion and 58 bp deletion, respectively.

To perform the targeted deletion of *ura3* in *G. lucidum* using the CRISPR/Cas9 system, *in vitro*‐transcribed sgRNA1 and sgRNA2 targeting *ura3* were mixed and transformed into protoplasts of the engineered *G. lucidum* pJW‐EXP‐intron‐opcas9 at a concentration of 100 µg (Fig. 4A). Figure 4B shows that numerous colonies appeared on the selective MM plate containing 5‐FOA after transformation. The transformants that were chosen continued to grow on the selective medium after three rounds of growth on a non‐selective medium. We characterized those transformants by genome PCR (Fig. 4C) and sequence analysis (Fig. 4D). The desired fragment deletion of *ura3* was found in transformants 4 and 5, demonstrating that the sequence between sgRNA1 and sgRNA2 can be deleted by CRISPR/Cas9‐mediated cleavage. The mutation frequency of the CRISPR/Cas9 system for each sgRNA was also determined by PCR and TA‐cloning. Primers ura3‐F and ura3‐R were used to detect the mutation of sgRNA1 and sgRNA2. A total of 30, 28 and 49 transformants from each genetic transformation were selected and then confirmed by sequence analysis. These results are summarized in Table 2 and Data S5. The average mutation frequency of *ura3* for sgRNA1 and sgRNA2 was 28.5% and 34.6%, respectively. The average frequency for dual sgRNA‐directed deletion (simultaneous mutation of two sites) of *ura3* was 36.7% in the engineered *G. lucidum* pJW‐EXP‐intron‐opcas9. These results illustrated that the CRISPR/Cas9 system is an effective tool for gene deletion in *G. lucidum*.

**Table 2:** The gene deletion frequencies of *ura3* in the pJW‐EXP‐intron‐opcas9 strain by dual sgRNA.

Target site	Number of mutants	Frequency (%)
sgRNA1	14 ± 2	28.5 ± 4
sgRNA2	17 ± 1	34.6 ± 2
sgRNA1 and sgRNA2	18 ± 3	36.7 ± 6

Forty‐nine individual 5‐FOA‐resistant mutants were assessed using the PCR and TA‐cloning sequence assay.

### Targeted deletion of the GL17624 gene in *G. lucidum* pJW‐EXP‐intron‐opcas9

**Figure 5:**
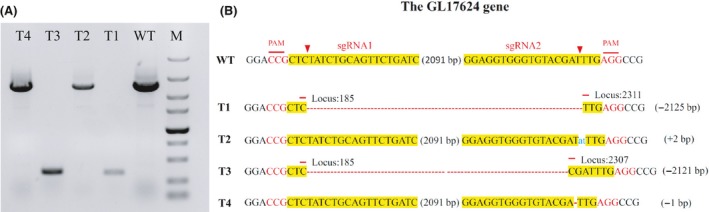
Dual sgRNA‐directed deletion of the GL 17624 gene in *G. lucidum*. A. Determination of the deletions of the GL 17624 gene in the *G. lucidum* transformants by PCR. B. TA‐cloning of the deletions of the GL 17624 gene in selected transformants. The sgRNA‐guiding sequences are highlighted in yellow. WT, the wild‐type strain; T, *G. lucidum* transformants; M, DL5000 DNA Marker

To investigate whether dual sgRNA‐directed gene deletion is applicable to other genomic loci in *G. lucidum*, the GL 17624 gene, which encodes a DNA‐binding protein, was targeted. We designed sgRNA1‐GL17624 and sgRNA2‐GL17624 (Data S3), which have targets 185 bp and 2311 bp downstream of the start codon of the GL 17624 gene, respectively. The two sgRNAs and the plasmid pJW‐EXP‐intron‐opbar were mixed and transformed into protoplasts of the engineered *G. lucidum* pJW‐EXP‐intron‐opcas9. Fifteen phosphinothricin‐resistant colonies were obtained in the selective CYM plate after 14 days of culture. The WT and all transformants were subjected to genome PCR analysis using the primers GL17624‐F and GL17624‐R (Table S1). The sequences of the PCR products are shown in Figure 5 and Data S6. The desired fragment deletion of the GL17624 gene was detected in 2 out of 15 transformants (13.3%). The PCR product showed a clear band for GL17624 (2469 bp) in the WT, but only an approximately 340 bp band was observed in the gene‐deleted transformants. The sequence analysis confirmed the expected deletion of the GL17624 gene in the obtained transformants. These results further validated that the use of the dual sgRNA‐directed CRISPR/Cas9 system is a promising approach for the deletion of target genes in *G. lucidum.*

## Discussion

**Figure 6:**
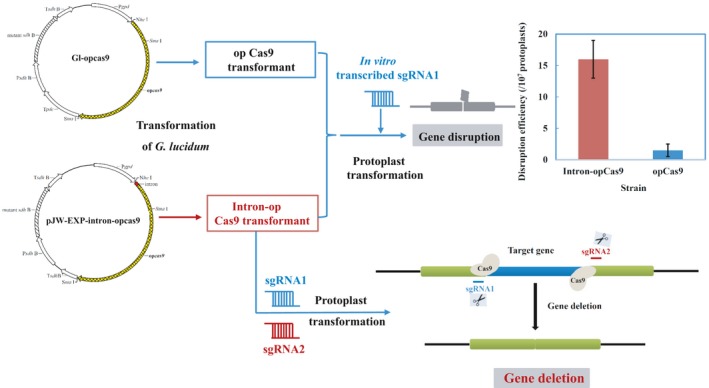
An effective platform for disruption and deletion of target genes in *G. lucidum*.

Although the CRISPR/Cas9 system has been used in the higher fungus *Ganoderma* for gene disruption (Qin *et al.*, 2017), its low disruption frequency hampered its application to genome engineering. We improved the frequency of gene disruption in *G. lucidum* by the introduction of an intron to the codon‐optimized Cas9. Furthermore, target gene deletion was achieved by employing the dual sgRNA‐directed CRISPR/Cas9 system in *G. lucidum* (Figure 6).

Phosphinothricin‐resistant and green fluorescent transformants were generated by adopting intron addition and codon optimization in *G. lucidum*. The results suggested that a 5′ intron was necessary for the efficient expression of heterologous genes in *G. lucidum.* Other heterologous genes may also be successfully expressed in *G. lucidum* by introducing an intron. This report agrees with previous reports on *Clitopilus passeckerianus*, *Phanerochaete chrysosporium* and *Agaricus bisporus*. The addition of an intron at the 5′ end of the phleomycin‐resistance gene was required for its efficient expression in *C. passeckerianus* (Kilaru *et al.*, 2009). The presence of a 5′ intron significantly affected the expression level of the enhanced GFP (EGFP) and GFP in *P. chrysosporium* and *A. bisporus* (Ma *et al.*, 2001; Burns *et al.*, 2005). An intron introduced before heterologous genes may increase the level of mRNA accumulation, thus upregulating protein expression in basidiomycete mushrooms. Previously, Mass et al. reported that the addition of the first intron of the maize Shrunken‐1 gene to constructs containing prokaryotic reporter genes dramatically increased the expression level of chloramphenicol transacetylase marker gene in monocotyledonous plants (Maas *et al.*, 1991). In *Schizophyllum commune*, the addition of an intron was sufficient to increase the mRNA accumulation of SC3 hydrophobin cDNA to a level similar to that of the genomic SC3 gene (Lugones *et al.*, 1999). Until now, only the carboxin and hygromycin resistance cassettes can be used in *G. lucidum* as a selectable marker. Now, the modified phosphinothricin‐resistance cassette is also available and is just as efficient for transformant selection. The expression of GFP will facilitate the determination of gene expression and protein localization in *G. lucidum*.

The gene disruption frequency significantly increased in the transformants when they contained the Cas9 gene with the *gpd* 5′ intron of *G. lucidum* compared with those in the transformant without the intron. Similar observations were also reported in potato and rice, in which insertion of a 5′‐untranslated region (UTR) upstream of the Cas9 gene elevated the mutation frequency of the target gene (Table 3). A previous study found that a large amount of transfected Cas9‐encoding mRNA increased gene mutation frequencies in zebrafish (Hwang *et al.*, 2013). In the liverwort *Marchantia polymorpha* L., the mutation frequency of the *ARF1* locus was increased by the strong promoter MpEPpro, thereby driving Cas9 gene expression (Sugano *et al.*, 2014). A positive correlation between the Cas9 expression level and CRISPR/Cas9‐mediated gene mutation frequency has also been reported in rice (Mikami *et al.*, 2015a, 2015b). The addition of an intron led to an increase in the transcription levels of the Cas9 gene (Fig. S3), resulting in an increased gene disruption frequency in *G. lucidum*. The engineered strain pJW‐EXP‐intron‐opcas9 is useful for efficient gene mutagenesis using the CRISPR/Cas9 system in *G. lucidum*.

**Table 3:** The effect of the insertion of a 5′‐UTR or intron upstream of the Cas9 gene on mutation frequencies.

Organism	Target gene	The insertion sequence	Fold increase in mutation frequency	Reference
Potato	*gbss1*	*OsMac3* 5′‐UTR	2.6	Kusano *et al.* (2018)
Rice	*ysa*	*adh2* 5′‐UTR	4.6	Mikami *et al.* (2015a)
*Ganoderma*	*ura3*	*gpd* intron 1	10.6	This study

The *ura3* and GL17624 gene fragments were successfully deleted in *G. lucidum* by applying the Cas9/dual sgRNA technology. To the best of our knowledge, this is the first description of target gene deletion in non‐model mushrooms using the dual sgRNA system. Our results suggest that this method is efficient for gene deletion in *G. lucidum*. The dual sgRNA system has also been used to delete target DNA fragments in rabbit and *indica* rice (Song *et al.*, 2016; Wang *et al.*, 2017). The application of dual sgRNAs would result in the simultaneous cleavage of the target gene by Cas9, leading to the elimination of the intervening fragment and ligation of both ends by DNA repair (Essletzbichler *et al.*, 2014). Our developed tool, based on dual sgRNA, is suitable for the deletion of target genes in basidiomycete mushrooms.

## Experimental procedures

### Strains and culture conditions

*Ganoderma lucidum* strain 260125 (a monokaryotic strain derived from the dikaryotic strain CGMCC 5.26) was used for CRISPR/Cas9 gene deletion experiments. This strain was maintained on potato dextrose agar slants. *Escherichia coli* strain DH5α was used in the construction of recombinant plasmids. *G. lucidum* cells were cultured in CYM plates (20 g l^−1^ glucose, 10 g l^−1^ maltose, 0.6 m mannitol, 2 g l^−1^ yeast extract, 2 g l^−1^ tryptone, 0.5 g l^−1^ MgSO_4_, 4.6 g l^−1^ KH_2_PO_4_ and 10 g l^−1^ agar) as described by Xu *et al.* (2012).

### Plasmid construction and *in vitro* transcription

All plasmids used were derived from the plasmid pJW‐EXP as a backbone. The pJW‐EXP was generated from the plasmid pMD19‐T by inserting the *G. lucidum gpd* promoter, the iron‐sulphur protein subunit of succinate dehydrogenase gene terminator, and the carboxin‐resistance gene as a selection marker (Yu *et al.*, 2014). The *bar* gene was acquired from the pBARGPE1 plasmid (University of Kansas Medical Center, Kansas City, KS, USA) using primers bar‐NheI‐F and bar‐SmaI‐R (Supplementary data, Table S1). This PCR fragment was digested with *Nhe*I and *Sma*I and ligated into *Nhe*I‐ and *Sma*I‐digested pJW‐EXP plasmids to yield pJW‐EXP‐bar. The plasmid pJW‐EXP‐opbar is similar to pJW‐EXP‐bar, except that *bar* was replaced by the codon‐optimized *bar* (*opbar*, Data S1), which was obtained from Shanghai Sangon Ltd., Crop. (Shanghai, China). The *opbar* was amplified using the primers opbar‐NheI‐F and opbar‐SmaI‐R (Supplementary data, Table S1). The pJW‐EXP plasmid was modified to produce pJW‐EXP‐intron‐opbar plasmid (5′ intron). It contained the *G. lucidum gpd* promoter sequence followed by the fragment intron (Data S2), which includes, from 5′ to 3′, *gpd*’s exon 1 (6 bp), intron 1 (67 bp), the 5′ end of exon 2 (3 bp) (Fei *et al.*, 2006), the *opbar* coding sequence, and the succinate dehydrogenase gene terminator T*sdh* B (Yu *et al.*, 2014). The fragment intron was cloned from *G. lucidum* genomic DNA by the primers gpd‐NheI‐Intron‐F and intron‐R (Supplementary data, Table S1). The *opbar* was cloned using the primers intron‐opbar‐F and ter‐SmaI‐opbar‐R (Supplementary data, Table S1). The fragment intron and the *opbar* were ligated into *Nhe*I‐ and *Sma*I‐digested pJW‐EXP plasmid using the ClonExpress MultiS one Step Cloning Kit (Vazyme, Nanjing, China) to generate the pJW‐EXP‐intron‐opbar plasmid.

The *gfp* and NheI‐Intron‐codon‐optimized GFP (*opgfp*)‐SmaI genes were ordered from Shanghai Sangon Ltd., Crop. (Shanghai, China). *Gfp* and *opgfp* were amplified from the synthetic sequences using the primers gfp‐NheI‐F/gfp‐SmaI‐R and opgfp‐NheI‐F/opgfp‐SmaI‐R (Supplementary data, Table S1), respectively. The fragments NheI‐gfp‐SmaI, NheI‐opgfp‐SmaI and NheI‐Intron‐opgfp‐SmaI were ligated into the *Nhe*I‐ and *Sma*I‐digested pJW‐EXP plasmid to produce the pJW‐EXP‐gfp, pJW‐EXP‐opgfp and pJW‐EXP‐intron‐opgfp plasmids, respectively.

The plasmid GL‐opcas9 was provided by Jian‐Jiang Zhong Laboratory. The plasmid pJW‐EXP‐intron‐opcas9 is similar to pJW‐EXP‐intron‐opbar, except that the *opbar* was replaced by the *opcas9*. The *opcas9* was amplified from plasmid GL‐opcas9 using the primers intron‐opcas9‐F and ter‐opcas9‐SmaI‐R (Supplementary data, Table S1). The fragment intron and *opcas9* were ligated into the *Nhe*I‐ and *Sma*I‐digested pJW‐EXP plasmid to produce the pJW‐EXP‐intron‐opcas9 plasmid using the ClonExpress MultiS one Step Cloning Kit (Vazyme, Nanjing, China).

The four sgRNA cassettes (Data S3), including two *ura3* and two GL26016 gene targeting sequences, and an sgRNA sequence were driven by a T7 promoter. The T7 promoter and four sgRNA cassettes were synthesized by Shanghai Sangon Ltd., Corp. (Shanghai, China). These sgRNA cassettes were transcribed *in vitro* using the HiScribe™ T7 High Yield RNA Synthesis Kit (NEB, Beijing, China) and purified using the RNA Clean & Concentrator™‐25 Kit (Zymo Research, Beijing, China).

### Genetic transformation of *G. lucidum*

The preparation of *G. lucidum* protoplasts and PEG‐mediated genetic transformation were conducted as previously described (Xu *et al.*, 2012, 2015; Yu *et al.*, 2014). Transformants were screened using carboxin (2 mg l^−1^) or phosphinothricin (150 mg l^−1^) on a CYM regeneration plate. For the protoplast transformation of sgRNA cassettes, the generated RNA (10 µg) was transformed into the opcas9 or the intron‐opCas9 transformant using the PEG‐mediated transformation procedure.

### Microscopic analysis for GFP expression

GFP expression was detected by microscopic screening with an excitation filter of 450‐490 nm, a dichroic filter of 510 nm and an emission filter of 515 nm as previously described (Ford *et al.*, 2016). Images were captured with a Nikon Coolpix 900 camera. Samples for microscopy were mycelia grown in a CYM plate for five days.

### Screening of *ura3* disruption or deletion mutants

Following the PEG‐mediated transformation of sgRNA cassettes targeting *ura3*, transformants were screened on an MM selection plate (20 g/L glucose, 2 g/L L‐asparagine, 0.6 m mannitol, 0.5 g l^−1^ MgSO_4_, 0.46 g l^−1^ KH_2_PO_4_, 1 g l^−1^ K_2_HPO_4_, 0.125 mg l^−1^ vitamin B_1_ and 10 g l^−1^ agar), including 400 mg l^−1^ 5‐FOA and 100 mg l^−1^ uridine (Sangon, Shanghai, China). The genomic DNA of the transformants was extracted using the Wizard Genomic DNA purification Kit (Promega, Beijing, China) for PCR amplification of *ura3* using the primers ura3‐F and ura3‐R (Supplementary data, Table S1). Gene disruption or deletion was confirmed using Sanger sequencing.

### Screening of the GL 17624 gene deletion mutants

The GL17624 gene of *G. lucidum* was acquired by genomic PCR using the primers GL17624‐F and GL17624‐R. Following the genetic transformation of protoplasts of the intron‐opCas9 strain with the pJW‐EXP‐intron‐opbar plasmid and the transcribed sgRNAs targeting the GL 17624 gene, transformants were picked from a CYM selection plate containing 150 mg l^−1^ phosphinothricin. The GL 17624 gene was amplified from the genomic DNA of transformants and the WT and sequenced to confirm the gene deletion.

## Conflicts of interest

None declared.

## Supporting information


**Data S1.** Sequences
**Data S2.** Sequences
**Data S3.** Sequences
**Data S4.** Sequences
**Table S1.** Primers used in the study
**Fig. S1.** Mycelia growth of the transformants GL‐opcas9 and pJW‐EXP‐intron‐opcas9 in CYM plates
**Fig. S2.** CRISPR/Cas9‐directed mutation of ura3 in G. lucidum. Alignments of ura3 mutants (M1, D1, In1, and In37) from 5‐FOA‐resistant colonies in sgRNA1 transformants. WT is the wild‐type strain ura3 from G. lucidum. The sgRNA1‐guiding sequence is highlighted in yellow. Replacements, deletions and insertions were found near the PAM sequence.
**Fig. S3.** Transcriptional levels of Cas9 gene in the GL‐opcas9 and pJW‐EXP‐intron‐opcas9 strains. Expression of Cas9 gene from the GL‐opcas9 strain is defined as 1.0, and expression levels in the pJW‐EXP‐intron‐opcas9 strain are displayed as fold increases over the reference sample. The following primers were used: Cas9‐f, 5’‐GAGGTCGCCTACCACGAGAAGT‐3’ and Cas9‐r, 5’ ‐TGGACGAGCTGGATGAAGAGC‐3’. * indicates statistical significance (P < 0.05) compared to the GL‐opcas9 strain (control).
