# Supplementary material for: Dual sgRNA‐directed gene deletion in basidiomycete Ganoderma lucidum using the CRISPR/Cas9 system
Source: Microb Biotechnol. 2020 Jan 20;13(2):386–96. doi: 10.1111/1751-7915.13534 (PMC7017817; doi:10.1111/1751-7915.13534)
Supplement: Supplementary file 1 — Data S1. Sequences Data S2. Sequences Data S3. Sequences Data S4. Sequences Table S1. Primers used in the study Fig. S1. Mycelia growth of the transformants GL‐opcas9 and pJW‐EXP‐intron‐opcas9 in CYM plates Fig. S2. CRISPR/Cas9‐directed mutation of ura3 in G. lucidum. Alignments of ura3 mutants (M1, D1, In1, and In37) from 5‐FOA‐resistant colonies in sgRNA1 transformants. WT is the wild‐type strain ura3 from G. lucidum. The sgRNA1‐guiding sequence is highlighted in yellow. Replacements, deletions and insertions were found near the PAM sequence. Fig. S3. Transcriptional levels of Cas9 gene in the GL‐opcas9 and pJW‐EXP‐intron‐opcas9 strains. Expression of Cas9 gene from the GL‐opcas9 strain is defined as 1.0, and expression levels in the pJW‐EXP‐intron‐opcas9 strain are displayed as fold increases over the reference sample. The following primers were used: Cas9‐f, 5’‐GAGGTCGCCTACCACGAGAAGT‐3’ and Cas9‐r, 5’ ‐TGGACGAGCTGGATGAAGAGC‐3’. * indicates statistical significance (P < 0.05) compared to the GL‐opcas9 strain (control). [file MBT2-13-386-s001.docx]

**Supplementary information, Data S1 Sequences**

**>*opbar***

ATGAGCCCAGAGCGTCGTCCTGCTGACATCCGCCGTGCTACCGAGGCTGACATGCCAGCGGTTTGCACCATCGTCAACCACTACATCGAGACGAGCACGGTCAACTTCCGCACCGAGCCCCAGGAGCCTCAGGAGTGGACGGATGACCTCGTCCGTCTGCGGGAGCGCTACCCCTGGCTCGTCGCCGAGGTCGACGGAGAGGTCGCCGGTATCGCTTACGCGGGTCCTTGGAAGGCCCGCAACGCCTACGATTGGACGGCCGAGTCTACCGTGTACGTCTCCCCCCGCCATCAACGGACCGGCCTTGGTTCCACCCTCTATACTCACCTGCTGAAGTCCCTCGAGGCCCAGGGCTTCAAGTCGGTGGTCGCGGTCATTGGGCTCCCCAACGACCCTTCGGTGCGCATGCACGAAGCGCTCGGCTATGCCCCCCGCGGCATGCTTCGCGCGGCCGGCTTCAAACACGGGAATTGGCACGACGTGGGCTTCTGGCAGCTTGACTTCTCGCTCCCCGTCCCGCCGCGCCCGGTCCTCCCCGTTACTGAAATTTGA

**>*gfp***

ATGAGTAAAGGAGAAGAACTTTTCACTGGAGTTGTCCCAATTCTTGTTGAATTAGATGGTGATGTTAATGGGCACAAATTTTCTGTCAGTGGAGAGGGTGAAGGTGATGCAACATACGGAAAACTTACCCTTAAATTTATTTGCACTACTGGAAAACTACCTGTTCCATGGCCAACACTTGTCACTACTTTCGGTTATGGTGTTCAATGCTTTGCGAGATACCCAGATCATATGAAACAGCATGACTTTTTCAAGAGTGCCATGCCTGAAGGTTATGTACAGGAAAGAACTATATTTTTCAAAGATGACGGGAACTACAAGACACGTGCTGAAGTCAAGTTTGAAGGTGATACCCTTGTTAATAGAATCGAGTTAAAAGGTATTGATTTTAAAGAAGATGGAAACATTCTTGGACACAAATTGGAATACAACTATAACTCACACAATGTATACATCATGGCAGACAAACAAAAGAATGGAATCAAAGTTAACTTCAAAATTAGACACAACATTGAAGATGGAAGCGTTCAACTAGCAGACCATTATCAACAAAATACTCCAATTGGCGATGGCCCTGTCCTTTTACCAGACAACCATTACCTGTCCACACAATCTGCCCTTTCGAAAGATCCCAACGAAAAGAGAGACCACATGGTCCTTCTTGAGTTTGTAACAGCTGCTGGGATTACACATGGCATGGATGAACTATACAAATAA

**>*opgfp***

ATGTCTAAGGGCGAGGAGCTCTTCACCGGCGTCGTCCCCATCCTCGTCGAGCTCGACGGCGACGTCAACGGCCACAAGTTCTCCGTCTCCGGCGAGGGCGAGGGCGACGCCACCTACGGCAAGCTCACCCTCAAGTTCATCTGCACCACCGGCAAGCTCCCCGTCCCCTGGCCCACCCTCGTCACCACCTTCGGCTACGGCGTCCAGTGCTTCGCCCGCTACCCCGACCACATGAAGCAGCACGACTTCTTCAAGTCCGCCATGCCCGAGGGCTACGTCCAGGAGCGCACCATCTTCTTCAAGGACGACGGCAACTACAAGACCCGCGCCGAGGTCAAGTTCGAGGGCGACACCCTCGTCAACCGCATCGAGCTCAAGGGCATCGACTTCAAGGAGGACGGCAACATCCTCGGCCACAAGCTCGAGTACAACTACAACTCCCACAACGTCTACATCATGGCCGACAAGCAGAAGAACGGCATCAAGGTCAACTTCAAGATCCGCCACAACATCGAGGACGGCTCCGTCCAGCTCGCCGACCACTACCAGCAGAACACCCCCATCGGCGACGGCCCCGTCCTCCTCCCCGACAACCACTACCTCTCCACCCAGTCGGCCCTCTCCAAGGACCCCAACGAGAAGCGCGACCACATGGTCCTCCTCGAGTTCGTCACCGCCGCCGGCATCACCCACGGCATGGACGAGCTCTACAAGTGA

**Supplementary information, Data S2 Sequences**

**>The fragment intron sequence**

ATGCCC *gtgagtcctgcatccccatcgtgcaccgtattcacctcatcgtttggcccccttctcacaggtcaag*

GTC

*gpd*’s extron 1 (6 bp) and the 5’ end of exon 2 (3 bp) was highlighted in red-color font.

**Supplementary information, Data S3 Sequences**

**>sgRNA1-*ura3* cassette**

TTCTAATACGACTCACTATAGGAGCAGAAGCCCCCTGCCAGTTTTAGAGCTAGAAATAGCAAGTTAAAATAAGGCTAGTCCGTTATCAACTTGAAAAAGTGGCACCGAGTCGGTGCTTTT

**>sgRNA2-*ura3* cassette**

TTCTAATACGACTCACTATAGGCCTCTTCCGTGTATGAGCGTTTTAGAGCTAGAAATAGCAAGTTAAAATAAGGCTAGTCCGTTATCAACTTGAAAAAGTGGCACCGAGTCGGTGCTTTT

**>sgRNA1-GL17624 gene cassette**

TTCTAATACGACTCACTATAGATCAGAACTGCAGATAGAGGTTTTAGAGCTAGAAATAGCAAGTTAAAATAAGGCTAGTCCGTTATCAACTTGAAAAAGTGGCACCGAGTCGGTGCTTTT

**>sgRNA2-GL17624 gene cassette**

TTCTAATACGACTCACTATAGGAGGTGGGTGTACGATTTGGTTTTAGAGCTAGAAATAGCAAGTTAAAATAAGGCTAGTCCGTTATCAACTTGAAAAAGTGGCACCGAGTCGGTGCTTTT

Target sequence of *ura3* and GL 26016 gene are underlined. T7 promoter sequence was highlighted in red-color font.

**Supplementary information, Data S3 Sequences**

**> Alignments of other 26 *ura3* mutants from 5-FOA-resistant colonies constitutive expression of Cas9 in sgRNA1 transformants. Replacement, deletion and insertion were found near the PAM sequence.**

SgRNA1

***ura3* sgRNA1**

WT：GTCGGTCTGCCCCTTGGCAGGGGGCTTCTGCTCCTCGCCGAGATGAGCACCAAGGGCAGCCTCG

1： GTCGGTCTGCCCCTTGGtCAGGGGGCTTCTGCTCCTCGCCGAGATGAGCACCAAGGGCAGCCTCG

2： GTCGGTCTGCCCCTTGGttCAGGGGGCTTCTGCTCCTCGCCGAGATGAGCACCAAGGGCAGCCTCG

3： GTCGGTCTGCCCCTTGGgtCAGGGGGCTTCTGCTCCTCGCCGAGATGAGCACCAAGGGCAGCCTCG

4： GTCGGTCTGCCCCTTGGtCAGGGGGCTTCTGCTCCTCGCCGAGATGAGCACCAAGGGCAGCCTCG

5： GTCGGTCTGCCCCTTGGtaaacgCAGGGGGCTTCTGCTCCTCGCCGAGATGAGCACCAAGGGCAGCC

6： GTCGGTCTGCCCCTTGGcgCAGGGGGCTTCTGCTCCTCGCCGAGATGAGCACCAAGGGCAGCCTCG

7： GTCGGTCTGCCCCTTGGcattCAGGGGGCTTCTGCTCCTCGCCGAGATGAGCACCAAGGGCAGCCTC

8： GTCGGTCTGCCCCTTGG**－**AGGGGGCTTCTGCTCCTCGCCGAGATGAGCACCAAGGGCAGCCTCG

9： GTCGGTCTGCCCCTTGGtatcttcgtctagagggCAGGGGGCTTCTGCTCCTCGCCGAGATGAGCACCAAGG

10：GTCGGTCTGCCCCTTGG**－－**GGGGGCTTCTGCTCCTCGCCGAGATGAGCACCAAGGGCAGCCTCG

11：GTCGGTCTGCCCCTTGGctagctagctatCAGGGGGCTTCTGCTCCTCGCCGAGATGAGCACCAAGGGCA

12：GTCGGTCTGCCCCTTGGtCAGGGGGCTTCTGCTCCTCGCCGAGATGAGCACCAAGGGCAGCCTCG

13：GTCGGTCTGCCCCTTGGgtgCAGGGGGCTTCTGCTCCTCGCCGAGATGAGCACCAAGGGCAGCCTC

14：GTCGGTCTGCCCCTTGGtacgcCAGGGGGCTTCTGCTCCTCGCCGAGATGAGCACCAAGGGCAGCCT

15：GTCGGTCTGCCCCTTGG**－**AGGGGGCTTCTGCTCCTCGCCGAGATGAGCACCAAGGGCAGCCTC

16：GTCGGTCTGCCCCTTGGtCAGGGGGCTTCTGCTCCTCGCCGAGATGAGCACCAAGGGCAGCCTCG

17：GTCGGTCTGCCCCTTGGtggCAGGGGGCTTCTGCTCCTCGCCGAGATGAGCACCAAGGGCAGCCTC

18：GTCGGTCTGCCCCTTGGgggCAGGGGGCTTCTGCTCCTCGCCGAGATGAGCACCAAGGGCAGCCTC

19：GTCGGTCTGCCCCTTGGtacCAGGGGGCTTCTGCTCCTCGCCGAGATGAGCACCAAGGGCAGCCTC

20：GTCGGTCTGCCCCTTGGgCAGGGGGCTTCTGCTCCTCGCCGAGATGAGCACCAAGGGCAGCCTCG

21：GTCGGTCTGCCCCTTGGttCAGGGGGCTTCTGCTCCTCGCCGAGATGAGCACCAAGGGCAGCCTCG

22：GTCGGTCTGCCCCTTGGtCAGGGGGCTTCTGCTCCTCGCCGAGATGAGCACCAAGGGCAGCCTCG

23：GTCGGTCTGCCCCTTGGatcCAGGGGGCTTCTGCTCCTCGCCGAGATGAGCACCAAGGGCAGCCTC

24：GTCGGTCTGCCCCTTGGtCAGGGGGCTTCTGCTCCTCGCCGAGATGAGCACCAAGGGCAGCCTCG

25：GTCGGTCTGCCCCTTGGttcgCAGGGGGCTTCTGCTCCTCGCCGAGATGAGCACCAAGGGCAGCCTC

26：GTCGGTCTGCCCCTTGGtCAGGGGGCTTCTGCTCCTCGCCGAGATGAGCACCAAGGGCAGCCTCG

**Supplementary information, Data S4 Sequences**

**> Alignments of other 28 *ura3* mutants from 5-FOA-resistant colonies constitutive expression of Cas9 in sgRNA2 transformants. Deletion and insertion were found near the PAM sequence.**

SgRNA2

***ura3* sgRNA2**

WT：GGCAGCCTCGCAACCGGCTCATACACGGAAGAGGCCGTCCGTATGGCTCGCGCCAACCGCGACT

1： GGCAGCCTCGCAACCGGCTttCATACACGGAAGAGGCCGTCCGTATGGCTCGCGCCAACCGCGAC

2： GGCAGCCTCGCAACCGGCTgtCATACACGGAAGAGGCCGTCCGTATGGCTCGCGCCAACCGCGAC

3： GGCAGCCTCGCAACCGGC**－－－**TACACGGAAGAGGCCGTCCGTATGGCTCGCGCCAACCGCGA

4： GGCAGCCTCGCAACCGGCTtactCATACACGGAAGAGGCCGTCCGTATGGCTCGCGCCAACCGCGA

5： GGCAGCCTCGCAACCGGCT**－**ATACACGGAAGAGGCCGTCCGTATGGCTCGCGCCAACCGCGACT

6： GGCAGCCTCGCAACCGGCTttggCATACACGGAAGAGGCCGTCCGTATGGCTCGCGCCAACCGCGA

7： GGCAGCCTCGCAACCGGCTgatCATACACGGAAGAGGCCGTCCGTATGGCTCGCGCCAACCGCGA

8： GGCAGCCTCGCAACCGGCT**－－**TACACGGAAGAGGCCGTCCGTATGGCTCGCGCCAACCGCGAC

9： GGCAGCCTCGCAACCGGCTtgttacctactCATACACGGAAGAGGCCGTCCGTATGGCTCGCGCCAACC

10： GGCAGCCTCGCAACCGGCTcactggtCATACACGGAAGAGGCCGTCCGTATGGCTCGCGCCAACCG

11： GGCAGCCTCGCAACCGGCT**－**ATACACGGAAGAGGCCGTCCGTATGGCTCGCGCCAACCGCGAC

12： GGCAGCCTCGCAACCGGCT**－**ATACACGGAAGAGGCCGTCCGTATGGCTCGCGCCAACCGCGAC

13： GGCAGCCTCGCAACCGGCTtacctaCATACACGGAAGAGGCCGTCCGTATGGCTCGCGCCAACCGC

14： GGCAGCCTCGCAACCGGCTaatCATACACGGAAGAGGCCGTCCGTATGGCTCGCGCCAACCGCGA

15： GGCAGCCTCGCAACCGGCTtCATACACGGAAGAGGCCGTCCGTATGGCTCGCGCCAACCGCGAC

16： GGCAGCCTCGCAACCGGCTtCATACACGGAAGAGGCCGTCCGTATGGCTCGCGCCAACCGCGAC

17： GGCAGCCTCGCAACCGGCTttCATACACGGAAGAGGCCGTCCGTATGGCTCGCGCCAACCGCGAC

18： G**－－－－－－－－－－－－－－－－－－－－－－－**CCGTCCGTATGGCTCGCGCCAACCGCGAC

19： GGCAGCCTCGCAACCGGCT**－**ATACACGGAAGAGGCCGTCCGTATGGCTCGCGCCAACCGCGAC

20： GGCAGCCTCGCAACCGGCTaaggtCATACACGGAAGAGGCCGTCCGTATGGCTCGCGCCAACCGC

21： GGCAGCCTCGCAACCGGCT**－**ATACACGGAAGAGGCCGTCCGTATGGCTCGCGCCAACCGCGAC

22： GGCAGCCTCGCAACCGGCT**－**ATACACGGAAGAGGCCGTCCGTATGGCTCGCGCCAACCGCGAC

23： GGCAGCCTCGCAACCGGCTccgaggaacatcacccctatCATACACGGAAGAGGCCGTCCGTATGGCTCGC

24： GGCAGCCTCGCAACCGGCT**－**ATACACGGAAGAGGCCGTCCGTATGGCTCGCGCCAACCGCGAC

25： GGCAGCCTCGCAACCGGCTtCATACACGGAAGAGGCCGTCCGTATGGCTCGCGCCAACCGCGAC

26： GGCAGCCTCGCAACCGGCTggtCATACACGGAAGAGGCCGTCCGTATGGCTCGCGCCAACCGCGA

27： GGCAGCCTCGCAACCGGCTttgaaaggacctCATACACGGAAGAGGCCGTCCGTATGGCTCGCGCCAA

28： GGCAGCCTCGCAACCGGCTctCATACACGGAAGAGGCCGTCCGTATGGCTCGCGCCAACCGCGAC

**Supplementary information, Data S5 Sequences**

**> Alignments of other 44 *ura3* mutants from 5-FOA-resistant colonies constitutive expression of Cas9 in sgRNA (sgRNA1 and sgRNA2) transformants. Deletion and insertion were found near the PAM sequence.**

SgRNA1 and SgRNA2

***ura3* sgRNA2**

***ura3* sgRNA1**

WT：TGCCCCTTGGCAGGGGGCTTCTGCTCCTCG（27bp）CAACCGGCTCATACACGGAAGAGGCCGTC

1：TGCCCCTTGGCAGGGGGCTTCTGCTCCTCG（27bp）CAACCGGCTgatgtaCATACACGGAAGAGGC

2：TGCCCCTTGG**－－－－－－－－－－－－－－－－－－－－－－－－－－－－－－－－**CATACACGGAAGAGGCCGTC

3：TGCCCCTTGGtCAGGGGGCTTCTGCTCCTCG（27bp）CAACCGGCTCATACACGGAAGAGGCCGTC

4：TGCCCCTTGG**－－－－－－－－－－－－－－－－－－－－－－－－－－－－－－－－**CATACACGGAAGAGGCCGTC

5：TGCCCCTTGG**－－－－－－－－－－－－－－－－－－－－－－－－－－－－－－－－**CATACACGGAAGAGGCCGTC

6：TGCCCCTTGG**－－－－－－－－－－－－－－－－－－－－－－－－－－－－－－－－**CATACACGGAAGAGGCCGTC

7：TGCCCCTTGGattCAGGGGGCTTCTGCTCCTCG（27bp）CAACCGGCT**－**ATACACGGAAGAGGCCGT

8：TGCCCCTTGG**－**AGGGGGCTTCTGCTCCTCG（27bp）CAACCGGCTCATACACGGAAGAGGCCGT

9：TGCCCCTTGGCAGGGGGCTTCTGCTCCTCG（27bp）CAACCGGCTggtCATACACGGAAGAGGCCGT

10：TGCCCCTTGG**－－－－－－－－－－－－－－－－－－－－－－－－－－－－－－－－**CATACACGGAAGAGGCCGTC

11：TGCCCCTTGG**－－－－－－－－－－－－－－－－－－－－－－－－－－－－－－－－**CATACACGGAAGAGGCCGTC

12：TGCCCCTTGG**－－－－－－－－－－－－－－－－－－－－－－－－－－－－－－－－**CATACACGGAAGAGGCCGTC

13：TGCCCCTTGGCAGGGGGCTTCTGCTCCTCG（27bp）CAACCGGCT**－**ATACACGGAAGAGGCCGTC

14：TGCCCCTTGGtgattgCAGGGGGCTTCTGCTCCTCG（27bp）CAACCGGCTCATACACGGAAGAGGCC

15：TGCCCCTTGG**－－－－－－－－－－－－－－－－－－－－－－－－－－－－－－－－**CATACACGGAAGAGGCCGTC

16：TGCCCCTTGGCAGGGGGCTTCTGCTCCTCG（27bp）CAACCGGCTggCATACACGGAAGAGGCCGT

17：TGCCCCTTGG**－－－－－－－－－－－－－－－－－－－－－－－－－－－－－－－－－**TACACGGAAGAGGCCGTC

18：TGCCCCTTGG**－**AGGGGGCTTCTGCTCCTCG（27bp）CAACCGGCTCATACACGGAAGAGGCCGTC

19：TGCCCCTTGGtCAGGGGGCTTCTGCTCCTCG（27bp）CAACCGGCTCATACACGGAAGAGGCCGTC

20：TGCCCCTTGGtcggCAGGGGGCTTCTGCTCCTCG（27bp）CAACCGGCTCATACACGGAAGAGGCCG

21：TGCCCCTTGGCAGGGGGCTTCTGCTCCTCG（27bp）CAACCGGCTctCATACACGGAAGAGGCCGT

22：TGCCCCTTGG**－－－－－－－－－－－－－－－－－－－－－－－－－－－－－－－－**CATACACGGAAGAGGCCGTC

23：TGCCCCTTGG**－－－－－－－－－－－－－－－－－－－－－－－－－－－－－－－－**CATACACGGAAGAGGCCGTC

24：TGCCCCTTGG**－－－－－－－－－－－－－－－－－－－－－－－－－－－－－－－－**CATACACGGAAGAGGCCGTC

25：TGCCCCTTGGcagatcCAGGGGGCTTCTGCTCCTCG（27bp）CAACCGGCTCATACACGGAAGAGGCC

26：TGCCCCTTGGtctccCAGGGGGCTTCTGCTCCTCG（27bp）CAACCGGCTCATACACGGAAGAGGCCG

27：TGCCCCTTGGCAGGGGGCTTCTGCTCCTCG（27bp）CAACCGGCTtaaagCATACACGGAAGAGGCC

28：TGCCCCTTGGCAGGGGGCTTCTGCTCCTCG（27bp）CAACCGGCT**－**ATACACGGAAGAGGCCGTC

29：TGCCCCTTGGCAGGGGGCTTCTGCTCCTCG（27bp）CAACCGG**－－－－**TACACGGAAGAGGCCG

30：TGCCCCTTGGCAGGGGGCTTCTGCTCCTCG（27bp）CAACCGGCTtgCATACACGGAAGAGGCCGT

31：TGCCCCTTGG**－－－－－－－－－－－－－－－－－－－－－－－－－－－－－－－－**CATACACGGAAGAGGCCGTC

32：TGCCCCTTGGCAGGGGGCTTCTGCTCCTCG（27bp）CAACCGGCTttggCATACACGGAAGAGGCCG

33：TGCCCCTTGGtCAGGGGGCTTCTGCTCCTCG（27bp）CAACCGGCTCATACACGGAAGAGGCCGTC

34：TGCCCCTTGG**－－－－－－－－－－－－－－－－－－－－－－－－－－－－－－－－**CATACACGGAAGAGGCCGTC

35：TGCCCCTTGGCAGGGGGCTTCTGCTCCTCG（27bp）CAACCGGCT**－**ATACACGGAAGAGGCCGTC

36：TGCCCCTTGGcaCAGGGGGCTTCTGCTCCTCG（27bp）CAACCGGCTCATACACGGAAGAGGCCGT

37：TGCCCCTTGGtCAGGGGGCTTCTGCTCCTCG（27bp）CAACCGGCTCATACACGGAAGAGGCCGTC

38：TGCCCCTTGG**－－－－－－－－－－－－－－－－－－－－－－－－－－－－－－－－－**TACACGGAAGAGGCCGTC

39：TGCCCCTTGGCAGGGGGCTTCTGCTCCTCG（27bp）CAACCGGCTtaCATACACGGAAGAGGCCGT

40：TGCCCCTTGG**－－－－－－－－－－－－－－－－－－－－－－－－－－－－－－－－**CATACACGGAAGAGGCCGTC

41：TGCCCCTTGGCAGGGGGCTTCTGCTCCTCG（27bp）CAACCGGCTtgttggCATACACGGAAGAGGCC

42：TGCCCCTTGGCAGGGGGCTTCTGCTCCTCG（27bp）CAACCGGCT**－－**TACACGGAAGAGGCCGTC

43：TGCCCCTTGGagccgCAGGGGGCTTCTGCTCCTCG（27bp）CAACCGGCTCATACACGGAAGAGGC

44：TGCCCCTTGGCAGGGGGCTTCTGCTCCTCG（27bp）CAACCGGCTtttCATACACGGAAGAGGCCGT

**Supplementary information, Data S6 Sequences**

**> Alignments of other GL17624 gene mutants. Deletion, insertion and inversion were found near the PAM sequence.**

**GL 17624 gene**

**GL17624 gene sgRNA1**

**GL17624 gene sgRNA2**

PAM sgRNA1 sgRNA2 PAM

WT: GGACCGCTCTATCTGCAGTTCTGATC (2091bp) GGAGGTGGGTGTACGATTTGAGGCCG

T5: GGACCGCTCggtTATCTGCAGTTCTGATC (2091bp) GGAGGTGGGTGTACGATTTGAGGCCG

T6: GGACCGCTCgTATCTGCAGTTCTGATC (2091bp) GGAGGTGGGTGTACGATttTTGAGGCCG

T7: GGACCGCT-TATCTGCAGTTCTGATC (2091bp) GGAGGTGGGTGTACGATTGAGGCCG

T8: GGACCGCTC--TCTGCAGTTCTGATC (2091bp) GGAGGTGGGTGTACGATTTGAGGCCG

T9: GGACCGCTCTATCTGCAGTTCTGATC (2091bp) GGAGGTGGGTGTA----TTGAGGCCG

T10: GGACCGCTCTATCTGCAGTTCTGATC (2091bp) GGAGGTGGGTGTACGATgtgTTGAGGCCG

T11: GGACCGCTCTAGCATGTGGGTGGAGG (2091bp inversion) CTAGTCTTGACGTCTATTTGAGGCCG

T12: GGACCGCTCcttTATCTGCAGTTCTGATC (2091bp) GGAGGTGGGTGTACG—TTGAGGCCG

T13: GGACCGCTCTATCTGCAGTTCTGATC (2091bp) GGAGGTGGGTGTACGATttgcTTGAGGCCG

T14: GGACCGCTC--TCTGCAGTTCTGATC (2091bp) GGAGGTGGGTGTA----TTGAGGCCG

T15:GGACCGCTCTATCTGCAGTTCTGATC(2091bp)GGAGGTGGGTGTACGATatagtagtgttaggtacatactctaaaaaattaaaaaaaagaaaaatTTGAGGCCG

**Supplementary information, Table S1**

**Primers used in the study**

| Name | Sequence (5’ to 3’) |
| --- | --- |
| bar-NheI-F | GCTAGCATGAGCCCAGAACGACGC |
| bar-SmaI-R | CCCGGGTTAGATCTCGGTGACGGGC |
| opbar-NheI-F | GCTAGCATGAGCCCAGAGCGTCGT |
| opbar-SmaI-R | CCCGGGTCAAATTTCAGTAACGGGGAGGAC |
| gpd-NheI-Intron-F | CATCCCCCTCTCAACGCTAGCATGCCCGTGAGTCCTGCA |
| intro-R | GACCTTGACCTGTGAGAAGGG |
| intro-opbar-F | TCACAGGTCAAGGTCAGCCCAGAGCGTCGTCC |
| ter-SmaI-opbar-R | CTCTGACCCGCTCATCCCGGGTCAAATTTCAGTAACGGGGAGGAC |
| gfp-NheI-F | GCTAGCGTGAGCAAGGGCGAGGAGCTG |
| gfp-SmaI-R | CCCGGGTTACTTGTACAGCTCGTCCATGC |
| opgfp-NheI-F | GCTAGCATGTCTAAGGGCGAGGAGCTCTTCACC |
| opgfp-SmaI-R | CCCGGGTCACTTGTAGAGCTCGTCCATGCC |
| intro-opcas9-F | TCACAGGTCAAGGTCATGGACAAGAAGTACAGCATCGG |
| ter-opcas9-SmaI-R | CTCTGACCCGCTCATCCCGGGTTACACCTTCCTCTTCTTCTTGGG |
| ura3-F | GAAACACGGTGGCACTTCAAT |
| ura3-R | CGTAAACACCCCGACCAACT |
| Gl17624-F | ATGCACGCATGGACCCACA |
| Gl17624-R | CTACCGTTCCGTCTTGCTCG |

**Supplementary information, Figure S1**

**
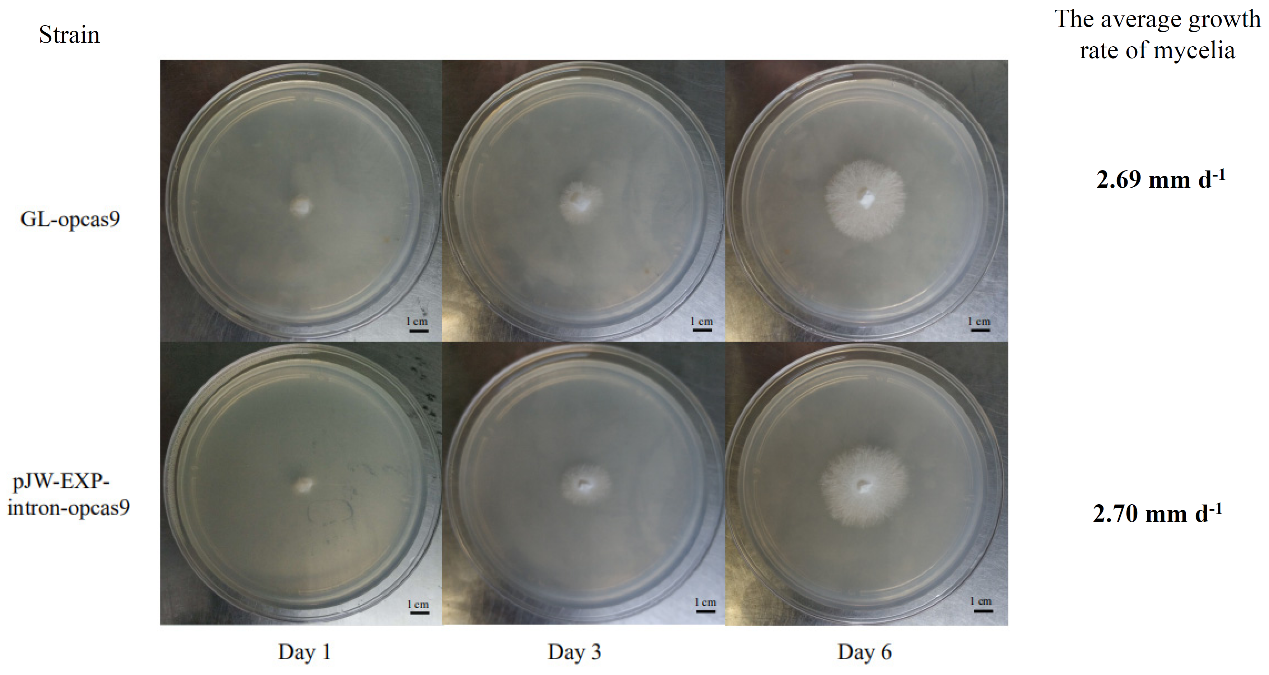
**

Mycelia growth of the transformants GL-opcas9 and pJW-EXP-intron-opcas9 in CYM plates

**Supplementary information, Figure S2**


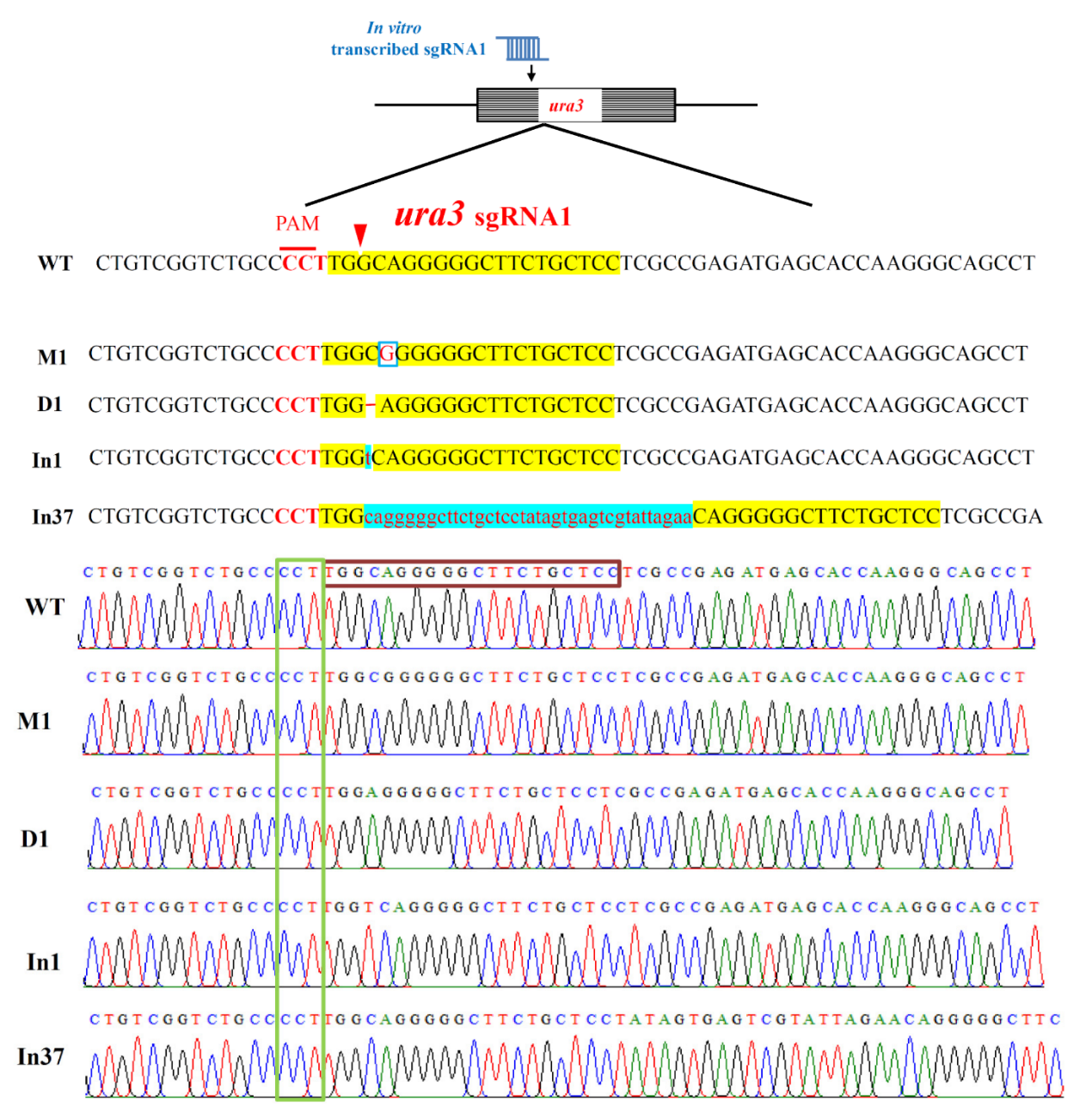


CRISPR/Cas9-directed mutation of *ura3* in *G. lucidum*. Alignments of *ura3* mutants (M1, D1, In1, and In37) from 5-FOA-resistant colonies in sgRNA1 transformants. WT is the wild-type strain *ura3* from *G. lucidum*. The sgRNA1-guiding sequence is highlighted in yellow. Replacements, deletions and insertions were found near the PAM sequence.

**Supplementary information, Figure S3**

**
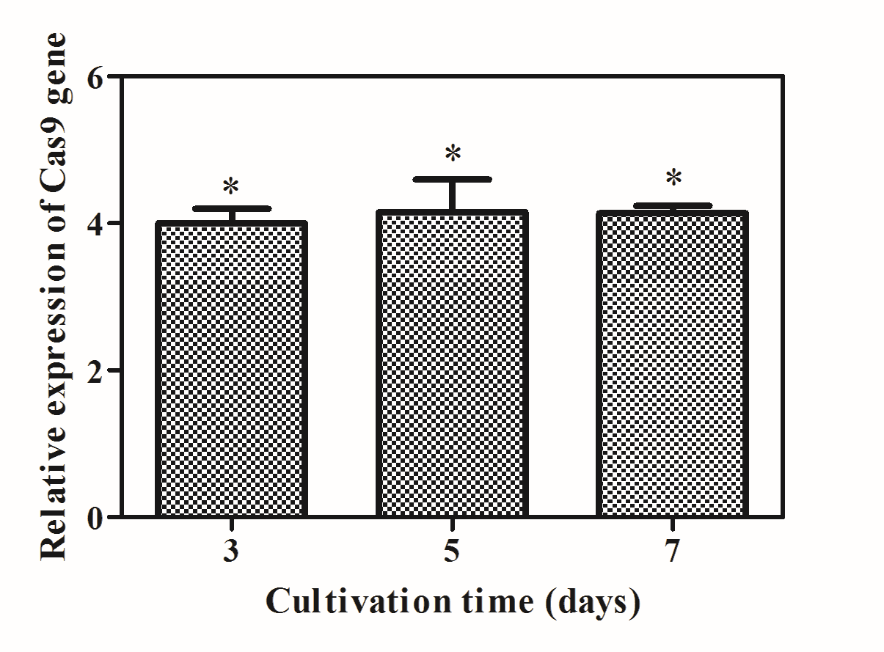
**

Transcriptional levels of Cas9 gene in the GL-opcas9 and pJW-EXP-intron-opcas9 strains. Expression of Cas9 gene from the GL-opcas9 strain is defined as 1.0, and expression levels in the pJW-EXP-intron-opcas9 strain are displayed as fold increases over the reference sample. The following primers were used: Cas9-f, 5’-GAGGTCGCCTACCACGAGAAGT-3’ and Cas9-r, 5’ -TGGACGAGCTGGATGAAGAGC-3’. * indicates statistical significance (P < 0.05) compared to the GL-opcas9 strain (control).
